# Estimating glomerular filtration in young people

**DOI:** 10.1093/ckj/sfae261

**Published:** 2024-08-28

**Authors:** Pierre Delanaye, Laurence Derain-Dubourg, Jonas Björk, Marie Courbebaisse, Lionel Couzi, Francois Gaillard, Cyril Garrouste, Anders Grubb, Lola Jacquemont, Magnus Hansson, Nassim Kamar, Christophe Legendre, Karin Littmann, Christophe Mariat, Lionel Rostaing, Andrew D Rule, Per-Ola Sundin, Arend Bökenkamp, Ulla Berg, Kajsa Åsling-Monemi, Anna Åkesson, Anders Larsson, Ulf Nyman, Hans Pottel

**Affiliations:** Department of Nephrology-Dialysis-Transplantation, University of Liège (ULg CHU), CHU Sart Tilman, Liège, Belgium; Department of Nephrology-Dialysis-Apheresis, Hopital Universitaire Caremeau, Nimes, France; Néphrologie, Dialyse, Hypertension et Exploration Fonctionnelle Rénale, Hôpital Edouard Herriot, Hospices Civils de Lyon, Lyon, France; Division of Occupational and Environmental Medicine, Lund University, Lund, Sweden; Clinical Studies Sweden, Forum South, Skåne University Hospital, Lund, Sweden; Physiology Department, Georges Pompidou European Hospital, Assistance Publique Hôpitaux de Paris, Paris Cité University, INSERM U1151-CNRS UMR8253, Paris, France; CHU de Bordeaux, Nephrologie – Transplantation – Dialyse, Université de Bordeaux, CNRS-UMR 5164 Immuno ConcEpT, Bordeaux, France; AURAL, Association pour l'utilisation du rein artificiel dans la région lyonnaise, Lyon, France; Department of Nephrology, Clermont-Ferrand University Hospital, Clermont-Ferrand, France; Department of Clinical Chemistry, Skåne University Hospital, Lund University, Lund, Sweden; Renal Transplantation Department, CHU Nantes, Nantes University, Nantes, France; Function area Clinical Chemistry, Karolinska University Laboratory, Karolinska University Hospital Huddinge and Department of Laboratory Medicine, Karolinska Institute, Stockholm, Sweden; Department of Nephrology, Dialysis and Organ Transplantation, CHU Rangueil, INSERM U1043, IFR – BMT, University Paul Sabatier, Toulouse, France; Hôpital Necker, AP-HP & Université Paris Descartes, Paris, France; Division of Clinical Chemistry, Department of Laboratory Medicine, Karolinska Institute, Huddinge, Sweden; Service de Néphrologie, Dialyse et Transplantation Rénale, Hôpital Nord, CHU de Saint-Etienne, Saint-Etienne, France; Service de Néphrologie, Hémodialyse, Aphérèses et Transplantation Rénale, Hôpital Michallon, CHU Grenoble-Alpes, Grenoble, France; Division of Nephrology and Hypertension, Mayo Clinic, Rochester, MN, USA; Karla Healthcare Center, Faculty of Medicine and Health, Örebro University, Örebro, Sweden; Department of Paediatric Nephrology, Emma Children's Hospital, Amsterdam UMC, Vrije Universiteit Amsterdam, Amsterdam, The Netherlands; Department of Clinical Science, Intervention and Technology, Division of Pediatrics, Karolinska Institutet, Karolinska University Hospital Huddinge, Stockholm, Sweden; Clinical Studies Sweden, Forum South, Skåne University Hospital, Lund, Sweden; Division of Occupational and Environmental Medicine, Lund University, Lund, Sweden; Clinical Studies Sweden, Forum South, Skåne University Hospital, Lund, Sweden; Department of Medical Sciences, Clinical Chemistry, Uppsala University, Uppsala, Sweden; Department of Translational Medicine, Division of Medical Radiology, Lund University, Malmö, Sweden; Department of Public Health and Primary Care, KU Leuven Campus Kulak Kortrijk, Kortrijk, Belgium

**Keywords:** creatinine, glomerular filtration rate, young adult

## Abstract

**Background:**

Creatinine-based equations are the most used to estimate glomerular filtration rate (eGFR). The Chronic Kidney Disease Epidemiology Collaboration (CKD-EPI), the re-expressed Lund-Malmö Revised (r-LMR) and the European Kidney Function Consortium (EKFC) equations are the most validated. The EKFC and r-LMR equations have been suggested to have better performances in young adults, but this is debated.

**Methods:**

We collected data (GFR) measured by clearance of an exogenous marker (reference method), serum creatinine, age and sex from 2366 young adults (aged between 18 and 25 years) both from Europe and the USA.

**Results:**

In the European cohorts (*n* = 1892), the bias (in mL/min/1.73 m²) was systematically better for the EKFC and r-LMR equations compared with the CKD-EPI equation [2.28, 95% confidence interval (1.59; 2.91), –2.50 (–3.85; –1.76), 17.41 (16.49; 18.47), respectively]. The percentage of estimated GFR within 30% of measured GFR (P30) was also better for EKFC and r-LMR equations compared with the CKD-EPI equation [84.4% (82.8; 86.0), 87.2% (85.7; 88.7) and 65.4% (63.3; 67.6), respectively]. In the US cohorts (*n* = 474), the bias for the EKFC and r-LMR equations was better than for the CKD-EPI equation in the non-Black population [0.97 (–1.69; 3.06), –2.62 (–5.14; –1.43) and 7.74 (5.97; 9.63), respectively], whereas the bias was similar in Black US individuals. P30 results were not different between the three equations in US cohorts. Analyses in sub-populations confirmed these results, except in individuals with high GFR levels (GFR ≥120 mL/min/1.73 m²) for whom the CKD-EPI equation might have a lower bias.

**Conclusions:**

We demonstrated that both the EKFC and r-LMR creatinine-based equations have a better performance than the CKD-EPI equation in a young population. The only exception might be in patients with hyperfiltration.

KEY LEARNING POINTS **What was known:**

- The best creatinine-based equation to be used in young people to estimate glomerular filtration rate (GFR) is still debated.

**This study adds:**

- We compare three equations [Chronic Kidney Disease Epidemiology Collaboration (CKD-EPI), the re-expressed Lund-Malmö Revised (r-LMR) and the European Kidney Function Consortium (EKFC)] in European and US cohorts in the largest study including young individuals.

**Potential impact:**

- We showed that EKFC and r-LMR equations were more accurate than the CKD-EPI equation to estimate GFR in young individuals both in Europe and USA.

## INTRODUCTION

Creatinine-based equations are still the most used equations to estimate glomerular filtration rate (eGFR) in clinical practice. Among these equations, the Chronic Kidney Disease Epidemiology Collaboration (CKD-EPI) [1], the Lund-Malmö Revised (LMR) [2, 3] and the European Kidney Function Consortium (EKFC) equations [4] are those that have been developed and validated with the largest datasets (Supplementary data, Table S1) [5–7]. These equations have been recently recognized as ‘validated’ by the Kidney Disease: Improving Global Outcomes (KDIGO) guidelines. The EKFC and the re-expressed version of LMR (r-LMR) equation [3] are both based on rescaled biomarkers and have been developed with the aim to avoid implausible jumps in eGFR at the transition between adolescence and adulthood. An implausible jump is something that is observed when separate equations are used for children and adults, notably with the Chronic Kidney Disease in Children (CKiD) and CKD-EPI equations [8]. It can be observed that serum creatinine in healthy populations is increasing during adolescence (because of growing and gain in muscular mass) and then remains quite constant from young adulthood to old ages, whereas GFR measured by a reference method is constant from 2 years to around 40 years, and then physiologically declines with aging [4, 9]. This observation was included in the development of the EKFC (based on rescaled serum creatinine, and using two splines with a knot at 40 years) [4] and in r-LMR (considering the rescaled serum creatinine concept) [3] equations, while the CKD-EPI equation assumes a constant decline across all ages, as it is based on regression of log(measured GFR) (mGFR) against log-(serum creatinine), sex and age [1, 6, 7]. This implies that CKD-EPI cannot be used in children due to massive overestimation of mGFR, and it also leads to overestimation in young adults of mGFR by the CKD-EPI equation which is observed both in European [4, 10, 11] and US cohorts [12]. Intriguingly, Inker *et al.* [13] recently published opposite findings for young adults. Indeed, in 1491 individuals aged between 18 and 40 years, they showed that the bias of the CKD-EPI equation was better [+0.5 (95% CI –0.7; 1.5) mL/min/1.73 m²] than the bias of EKFC [–4.9 (95% CI –5.7; –3.7) mL/min/1.73 m²]. However, accuracy within 30% (P30) (the percentage of eGFR within 30% of mGFR) remained very similar [88.9% (87.3; 90.5) versus 89.7% (88.3; 91.3)]. Focusing on the 276 patients aged between 18 and 25 years of age, the difference in bias was still more impressive [+3.3 (95% CI 0.0; 5.0) mL/min/1.73 m² versus –11.0 (95% CI –13.6; –7.3) mL/min/1.73 m² for the CKD-EPI and EKFC equations, respectively], whereas P30 was the same for both equations [90.2% (86.6; 93.5)].

In the Inker study, the sample size of individuals aged between 18 and 25 years was, however, relatively small (*n* = 276). The sample size available to the EKFC in the same age range (18–25 years) is much larger (*n* = 2366), allowing several subgroup analyses. Focusing on the young population (18–25 years), the aim of the present study was thus to compare the performance of r-LMR, EKFC and CKD-EPI equations in European and US cohorts.

## MATERIALS AND METHODS

The equations used in the current analysis are described in Supplementary data, Table S1. We only considered the race-free CKD-EPI equation [1, 14]. We here present results from two datasets including subjects aged from 18 to 25 years. The one from Europe was previously used for the seminal article of the EKFC equation, but excluding data from USA [4]. All participants were considered as White in this dataset [4]. We also used data obtained from US cohorts that have been described elsewhere [12]. Briefly, in all European cohorts, creatinine was directly measured with an IDMS traceable assay, whereas serum creatinine results were mostly indirectly recalibrated in US cohorts [4, 12, 15]. GFR was measured by a recognized reference method [16, 17]. Characteristics of the cohorts are described in Supplementary data, Table S2.

All analyses and calculations were performed using SAS 9.4 (SAS Institute Inc., Cary, NC, USA). Data were presented as mean ± standard deviation (SD) when the distribution was normal and as median with interquartile range (quartile 1–quartile 3) when not. Normality was assessed using the Shapiro–Wilk test. Performance of GFR equations was compared with usual metrics: median bias (i.e. eGFR – mGFR) with 95% confidence intervals (CI) which is the systematic difference between estimation and measurement, imprecision (interquartile range of the bias) which is the random difference between estimation and measurement, as well as P30 and P20 accuracy (percentage of eGFR values within ±30% or 20% of mGFR) with 95% CI. The target for bias was zero, but an absolute bias of at most 5 mL/min/1.73 m² might be considered reasonable. Imprecision should be as low as possible [18]. The goal for P30 was 100%, yet P30 >75% has been considered as ‘sufficient for good clinical decision making’ [19]. To test whether an equation is different from another equation in the same population, we did not use statistical tests to avoid numerous *P*-value calculations, but we considered an equation as different when the 95% CI between equations was not overlapping, which is a more conservative criterion. Median bias across the age spectrum was graphically presented using median quantile regression with 4th degree polynomials. We used all available data from the 18–100 years age range to calculate median quantiles, but here present the median quantile line restricted to the young adult age range [4, 12]. Likewise, accuracy P30 (%) was graphically presented across the age spectrum using cubic splines (3rd degree polynomial) with three free knots. Again, all available data were used for this calculation, but the P30 graph was restricted to the young adult age range. We have also presented the classical Bland and Altman figures to illustrate the performance of the equations according to GFR (expressed in an absolute or relative way).

We did a subgroup analysis according to sex, race (only in the US), eGFR (as usually considered by the CKD-EPI group) or mGFR (as usually considered by the EKFC) levels [20, 21], and using the usual categories (<30, 30–45, 45–60, 60–90, 90–120 and ≥120 mL/min/1.73 m²). We also performed an analysis according to body mass index (BMI), but only in European cohorts as there were too many missing data for height (*n* = 343) in the US cohorts.

The EKFC equation uses the concept of rescaled creatinine, which is the creatinine of the subject rescaled by a Q-value which is corresponding to the median serum creatinine concentration observed in a given population [6, 7]. Between 2 and 25 years, the Q-value is a polynomial calculation based on age: for men: ln(Q) = 3.200 + 0.259 × age –0.543 × log(age) – 0.00763 × age² + 0.0000790 × age³, and for women: ln(Q) = 3.080 + 0.177 × age – 0.223 × log(age) – 0.00596 × age² + 0.0000686 × age³ (serum creatinine being expressed in µmol/L in this equation). These polynomials level off at 0.70 mg/dL for females and 0.90 mg/dL for males, at the age of 25 years [4]. The race-free US Q-values have been previously established and validated, as a fixed value (0.97 mg/dL in males and 0.73 mg/dL in females) [12, 22, 23]. However, race-free US Q-values before 25 years were here calculated, as follows. The basic assumption is that the serum creatinine growth curves of White Europeans serve as a model but a gradual change is obtained between 12 years (assuming all healthy children have the same average serum creatinine until 12 years) and 25 years, by applying a linear relationship for the multiplication factor between ‘1’ (at 12 years) and the ratio Q-new population/Q-Europe. Consequently, all ‘healthy’ children at 12 years are supposed to have the same ideal serum creatinine value (Q-value), which gradually changes to the plateau value at 25 years, corresponding to the specific population.

We also compared the performance of the EKFC equation with the polynomial Q-values, as it was developed in the seminal EKFC article with results using a fixed Q-values as used in individuals older than 25 years (i.e. 0.7 mg/dL for females and 0.9 mg/dL in males).

## RESULTS

**Figure 1:**
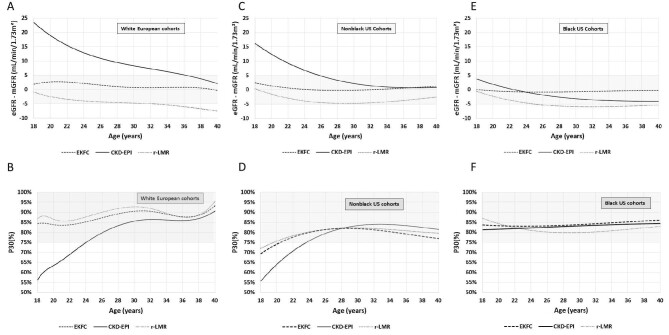
(**A**–**F**) Bias and P30 for the different equations according to age in the European, non-Black US and Black US cohorts. The grey area indicates the region where bias was zero ±5 mL/min/1.73 m² or P30 was ≥75%.

**Figure 2:**
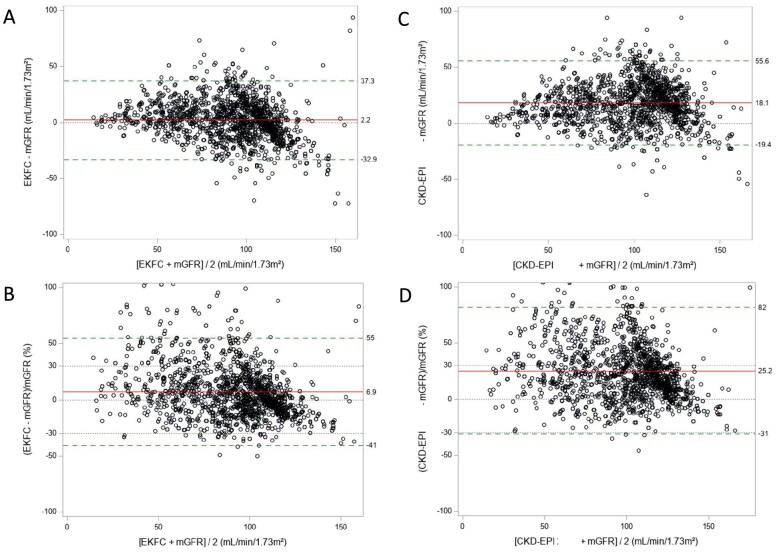
The Bland and Altman plots [absolute (**A, C**) or relative (**B, D**) bias] in the European cohorts for the EKFC (A, B) and CKD-EPI equations (C, D). The red line is the bias (mean difference) and the limits of agreement are between the green dotted lines. The grey dotted lines are corresponding to the zero bias line and the ±30% limits.

**Figure 3:**
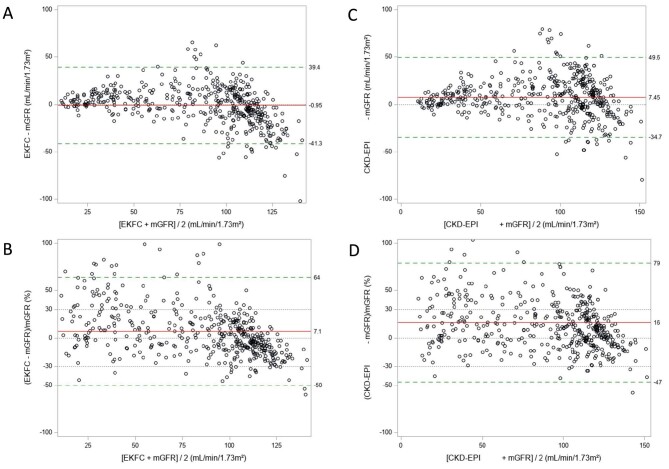
The Bland and Altman plots [absolute (**A, C**) or relative (**B, D**) bias] in the non-Black US cohorts for the EKFC (A, B) and CKD-EPI equations (C, D). The red line is the bias (mean difference) and the limits of agreement are between the green dotted lines. The grey dotted lines are corresponding to the zero bias line and the ±30% limits.

The sample size from European and US cohorts was 1892 and 474, respectively. Mean age was 20.7 ± 2.1 and 22.2 ± 2.2 (range 18–25) years, mean mGFR was 89.5 ± 28.9 mL/min/1.73 m² and 61.5 ± 35.3 mL/min/1.73 m², respectively (Supplementary data, Table S2). In the European cohorts (Table 1), bias and P30 were systematically better for the EKFC and r-LMR equations compared with the CKD-EPI equation, which largely overestimated mGFR (Table 1). r-LMR and EKFC equations performed similarly. In the US cohorts, the bias for the EKFC and r-LMR equations was also better than for the CKD-EPI equation in the whole and non-Black population, whereas the bias was similar in Black US individuals. P30 results were not different between the three equations in the US population. The bias and P30 results for the EKFC, r-LMR and CKD-EPI equations according to age in the White European cohorts are shown in Fig. 1A and B, in the non-Black US cohorts in Fig. 1C and D, and in the Black US cohorts in Fig. 1E and F. The Bland and Altman plots [with absolute bias (Fig. 1A) or relative bias (Fig. 1B)] confirmed the overestimation of the CKD-EPI equation, both in White Europeans (Fig. 2A and B) and US non-Black populations (Fig. 3).

**Table 1:** Performance of the CKD-EPI and EKFC equations between 18 and 25 years old.

		Bias			IQR			P30		
	*n*	CKD-EPI	EKFC	r-LMR	CKD-EPI	EKFC	r-LMR	CKD-EPI	EKFC	r-LMR
Europe	1892	17.41 (16.49; 18.47)	**2.28 (1.59; 2.91)**	**–2.50 (–3.85; –1.76)**	23.5 (6.0; 29.5)	21.4 (–8.7; 12.7)	21.9 (–14.2; 7.7)	65.4 (63.3; 67.6)	**84.4 (82.8; 86.0)**	**87.2 (85.7; 88.7)**
USA	474	7.02 (4.81; 8.96)	**0.37 (–1.69; 2.24)**	**–2.85 (–5.08; –1.65)**	22.5 (–3.0; 19.5)	22.3 (–11.8; 10.5)	24.3 (–17.7; 6.5)	74.1 (70.1; 78.0)	79.3 (75.7; 83.0)	80.4 (76.8; 84.0)
USA non-Black	437	7.74 (5.97; 9.63)	**0.97 (–1.69; 3.06)**	**–2.62 (–5.14; –1.43)**	23.1 (–3.0; 19.5)	23.1 (–12.0; 11.2)	24.9 (–18.0; 6.9)	73.2 (69.1; 77.4)	78.9 (75.1; 82.8)	79.9 (76.1; 83.6)
USA Black	37	0.05 (–2.71; 2.04)	–0.78 (–5.47; 0.80)	–3.62 (–6.81; –0.99)	13.8 (–3.7; 10.1)	9.3 (–6.7; 2.6)	10.1 (–9.2; 1.0)	83.8 (71.9; 95.7)	83.8 (71.9; 95.7)	86.5 (75.5; 97.5)

Bias and IQR are expressed in mL/min/1.73m². P30 is expressed in %.

Significant better results are in **bold**.

IQR: interquartile range (of the bias); NA: not available.

In the analysis according to sex (Supplementary data, Table S3), the same conclusions as in the whole population can be drawn in the European cohort. In US cohorts, only the bias in males was better for EKFC and r-LMR than for CKD-EPI, and all other results were similar.

In the US cohorts, the analysis according to race is shown in Table 1, but the sample size of Black individuals was low. The bias for the EKFC equation was better than for the CKD-EPI equation in non-Black individuals, whereas other metrics were not different. EKFC and CKD-EPI had the same performance in Black populations.

In the analysis stratified by GFR (Supplementary data, Table S4), EKFC and r-LMR had the same performance (with some exceptions favouring EKFC or r-LMR). In European cohorts, bias and P30 for EKFC and r-LMR were similar and frequently better than for CKD-EPI, whether the stratification was on eGFR or mGFR. The only exception was in the stratification on mGFR in the subgroup with mGFR ≥120 mL/min/1.73 m² (*n* = 256), where both the bias and the P30 for the CKD-EPI equation was better than for the EKFC or r-LMR equations.

In US cohorts, the performance of the three equations was similar, except in some groups where EKFC and r-LMR were better. Once again, CKD-EPI was better than EKFC in the stratification on mGFR only in the subgroup with mGFR ≥120 mL/min/1.73 m² (*n* = 93).

In the analysis according to BMI (Supplementary data, Table S5), the same conclusions as in the whole population can be drawn in the European cohort.

Considering the polynomials Q-values versus fixed Q results, we observed little impact on the global performance of the EKFC equations, as only the bias was better with the polynomial Q-value in the European whole cohort (Supplementary data, Table S6).

Because an equation performs better in the cohorts that were used for its development, we repeated analyses in the European participants who were in the external validation cohort of the EKFC equation [i.e. subjects from Amsterdam, France (Kidney Donors Study), Leuven and Lund, *n* = 240]. We observed a better bias for EKFC compared with the CKD-EPI equation [+5.25 (2.41; 6.64) versus +19.53 (16.87; 21.78) mL/min/1.73 m², respectively), and a better P30 result [87.1 (82.8; 91.3) versus 64.4 (59.4; 71.4) %, respectively].

## DISCUSSION

The new creatinine-based EKFC equation has been recently considered as a validated equation to estimate GFR by the KDIGO guidelines [24]. Moreover, this equation has been shown to have better performance than the CKD-EPI equation by different authors in different populations [25–27], but few focused on young populations. The better performance of the EKFC equation over the CKD-EPI has recently been questioned in young adults [13]. However, in the present analysis, we confirmed that both the EKFC and r-LMR equations have a significant better performance (both bias and accuracy) than the CKD-EPI equation, which overestimates GFR in this age range. This is one of the main advantages of the EKFC and r-LMR equations—having a better performance in young adults, allowing a smooth transition in estimating GFR between children, adolescents and young adults—whereas between 40 and 65 years, EKFC, r-LMR and CKD-EPI are quite equivalent [4, 6–8]. The theoretical advantage of the EKFC compared with the CKD-EPI equation in young adults is illustrated in the Supplementary data, Figs S1–S4. To the best of our knowledge, our analysis is the largest ever conducted in this age range (*n* = 2366), with data coming from both Europe and USA. In Europe, the performance advantage of EKFC and r-LMR is obvious, even if it should be recognized that some cohorts studied here have been used for the development of the EKFC equation [3, 4, 28]. However, we showed that the better performance was also observed in the external validation cohorts used in the seminal article on the EKFC equation. In the US cohorts, the performance of the EKFC and r-LMR equations is better than that of the CKD-EPI in young people (bias closer to zero and higher P30 values), at least in non-Black individuals. This result is remarkable as many US cohorts in the current analysis have been used in the development and/or validation of the CKD-EPI equation [12]. From our subgroup analysis, we learn that there is only one specific subgroup in which the CKD-EPI equation has a better bias (both in European and US cohorts) and P30 (in European cohorts) and this is in patients with mGFR >120 mL/min/1.73 m², corresponding to individuals with some degree of hyperfiltration [29, 30]. This result is of importance and can, at least in part, explain the discrepant conclusions from the analysis performed by Inker *et al.* in young adults [13]. Indeed, in their recent article, the mean mGFR in the individuals aged from 18 to 25 years is high, with a median of 110.0 [93.0;126.9] or a mean of 106.8 ± 33.0 mL/min/1.73 m² (these results were much higher than the mGFR observed in other age groups younger than 40 years old in the same analysis), assuming that a large part of these individuals were well hyperfiltrating [13]. Moreover, it should be mentioned that in cohorts used in Inker's studies (Table S1 in [13]), two cohorts (Renin Angiotensin System Study, *n* = 192 and Interdiabetes study, *n* = 16) had a particularly low mean age (28.6 ± 6.3 and 23.9 ± 5.7 years) and both were type 1 diabetic patients with a large proportion of hyperfiltrating individuals (mean mGFR at 131.2 ± 19.5 and 149.4 ± 21.6 mL/min/1.73 m²). Therefore, the conclusion of Inker *et al.* that the CKD-EPI equation is better than the EKFC (or r-LMR) equation in young people might only be correct in the very specific group of patients who are hyperfiltrating (GFR ≥120 mL/min/1.73 m²), where two estimations errors in opposite directions tend to cancel out. Moreover, another explanation can also be suggested when serum creatinine concentrations in the Inker study are examined. Indeed, for the same (hyperfiltrating) individuals aged between 18 and 25 years, mean serum creatinine concentration in this study was quite high 1.00 ± 0.80 mg/dL [13]. The high SD value also suggests non-normality (probably skewed to the right), indicating that many individuals even have higher serum creatinine concentrations than 1.00 mg/dL, which is not expected in individuals with such high mGFR levels. Once again, the majority of patients come from the two cohorts of patients with diabetes. The way serum creatinine has been calibrated in these two cohorts is obscure [15, 31–35]. Suspicious and abnormally high serum creatinine in the cohorts considered in the Inker study [13] will normally lead to underestimation of GFR, which could be here counterbalanced by the overestimation of CKD-EPI eGFR, usually seen in the age range we focused on in the current analysis [4, 6, 7, 12].

We also showed that in this specific age range (18–25 years), using the polynomial Q-value or the fixed Q-value as this is the case in individuals over 25 years [4] has little impact on the accuracy of the EKFC equation. This is correct for a cross-sectional analysis such as the current one. However, we must keep in mind that one major advantage of the EKFC (and r-LMR) equation is that it retains continuity in estimating GFR during the transition between childhood and adulthood, and in this context, using the polynomial Q-value still makes sense [4, 6, 8, 12].

Our study is not without limitations. First, some subanalyses include only a small number of individuals, and these results should thus be interpreted with caution. Second, only non-Black individuals are included in the European cohorts, and further studies are required in African, Asian and non-White European populations. In addition, the percentage of Black individuals from US cohorts is low in young US cohorts, but this limitation is also shared by the CKD-EPI group (*n* = 24 in [13]). Also, the European cohort presented here has been used to develop or validate the EKFC equation [4], and an equation usually performs well in the cohort used for its development. However, the different subanalyses were all original and relevant to confirm the superiority of the EKFC equation in young people. It was also important to perform a fair comparison between US and European cohorts. Finally, cystatin C results were not available in US cohorts, and the interest of this biomarker in young people needs to be studied in detail in future.

In the current analysis, we demonstrated that both the EKFC and r-LMR creatinine-based equations have a better performance than the CKD-EPI equation in a young population. The only exception might be in patients with hyperfiltration.

## Supplementary Material

sfae261_Supplemental_File

## Data Availability

Statistical code: the SAS code is available to interested readers by contacting H.P. at hans.pottel@kuleuven.be.

## Notes

The EKFC data set used in the present study is hosted by the Lund University Population Research Platform. Legal and ethical restrictions prevent public sharing of the data set. Data may be made available to interested researchers for collaboration upon request but would generally require a new ethical permission and the permission of each of the data owners. Contact information for the data host may be found at www.lupop.lu.se. The data from the MDRD, AASK, ALTOLD, CRIC, CRISP, DCCT/EDIC and PERL studies reported here are available upon request in the NIDDK Central Repository. The data from the Mayo Clinic are not publicly available due to the confidential nature of patient information obtained for clinical care. Legal and ethical restrictions prevent public sharing of the dataset. Data can be made available for collaborations upon request to interested researchers but would generally require a new ethical permission and the permission of each of the data owners.

## References

[1] Inker LA, Eneanya ND, Coresh J et al. New creatinine- and cystatin C-based equations to estimate GFR without race. N Engl J Med 2021;385:1737–49. 10.1056/NEJMoa2102953 34554658 PMC8822996

[2] Björk J, Grubb A, Sterner G et al. Revised equations for estimating glomerular filtration rate based on the Lund-Malmö Study cohort. Scand J Clin Lab Invest 2011;71:232–9. 10.3109/00365513.2011.557086 21391777

[3] Nyman U, Björk J, Delanaye P et al. Rescaling creatinine makes GFR estimation equations generally applicable across populations—validation results for the Lund-Malmö equation in a French cohort of sub-Saharan ancestry. Clin Chem Lab Med 2024;62:421–7. 10.1515/cclm-2023-0496 37768854

[4] Pottel H, Björk J, Courbebaisse M et al. Development and validation of a modified full age spectrum creatinine-based equation to estimate glomerular filtration rate. A cross-sectional analysis of pooled data. Ann Intern Med 2021;174:183–91. 10.7326/M20-4366 33166224

[5] Delanaye P, Cavalier E, Pottel H et al. New and old GFR equations: a European perspective. Clin Kidney J 2023;16:1375–83. 10.1093/ckj/sfad039 37664574 PMC10469124

[6] Delanaye P, Pottel H, Cavalier E et al. Diagnostic standard: assessing glomerular filtration rate. Nephrol Dial Transplant 2024;39:1088–96. 10.1093/ndt/gfad241 37950562

[7] Delanaye P, Cavalier E, Stehlé T et al. Glomerular filtration rate estimation in adults: myths and promises. Nephron 2024;148:408–14. 10.1159/000536243 38219717

[8] Pottel H, Björk J, Bökenkamp A et al. Estimating glomerular filtration rate at the transition from pediatric to adult care. Kidney Int 2019;95:1234–43. 10.1016/j.kint.2018.12.020 30922665

[9] Pottel H, Vrydags N, Mahieu B et al. Establishing age/sex related serum creatinine reference intervals from hospital laboratory data based on different statistical methods. Clin Chim Acta 2008;396:49–55. 10.1016/j.cca.2008.06.017 18621041

[10] Delanaye P, Vidal-Petiot E, Björk J et al. Performance of creatinine-based equations to estimate glomerular filtration rate in White and Black populations in Europe, Brazil, and Africa. Nephrol Dial Transplant 2023;38:106–18. 10.1093/ndt/gfac241 36002032

[11] Pottel H, Björk J, Rule AD et al. Cystatin C-based equation to estimate GFR without the inclusion of race and sex. N Engl J Med 2023;388:333–43. 10.1056/NEJMoa2203769 36720134

[12] Delanaye P, Rule AD, Schaeffner ES et al. Performance of the European kidney function consortium (EKFC) creatinine-based equation in American cohorts. Kidney Int 2024;105:629–37. 10.1016/j.kint.2023.11.024 38101514

[13] Inker LA, Tighiouart H, Adingwupu OM et al. Performance of GFR estimating equations in young adults. Am J Kidney Dis 2024;83:272–6. 10.1053/j.ajkd.2023.06.008 37717845 PMC11080956

[14] Delgado C, Baweja M, Crews DC et al. A unifying approach for GFR estimation: recommendations of the NKF-ASN Task Force on reassessing the inclusion of race in diagnosing kidney disease. J Am Soc Nephrol 2021;32:2994–3015. 10.1681/ASN.2021070988 34556489 PMC8638402

[15] Pottel H, Cavalier E, Björk J et al. Standardization of serum creatinine is essential for accurate use of unbiased estimated GFR equations: evidence from three cohorts matched on renal function. Clin Kidney J 2022;15:2258–65. 10.1093/ckj/sfac182 36381377 PMC9664577

[16] Soveri I, Berg UB, Björk J et al. Measuring GFR: a systematic review. Am J Kidney Dis 2014;64:411–24. 10.1053/j.ajkd.2014.04.010 24840668

[17] Delanaye P, Ebert N, Melsom T et al. Iohexol plasma clearance for measuring glomerular filtration rate in clinical practice and research: a review. Part 2: why to measure glomerular filtration rate with iohexol? Clin Kidney J 2016;9:682–99. 10.1093/ckj/sfw070 27679715 PMC5036902

[18] Delanaye P, Pottel H, Botev R. Con: should we abandon the use of the MDRD equation in favour of the CKD-EPI equation? Nephrol Dial Transplant 2013;28:1396–403. 10.1093/ndt/gft006 23780677

[19] National Kidney Foundation . K/DOQI clinical practice guidelines for chronic kidney disease: evaluation, classification, and stratification. Am J Kidney Dis 2002;39:S1–266. 11904577

[20] Björk J, Grubb A, Sterner G et al. Performance of GFR estimating equations stratified by measured or estimated GFR: implications for interpretation. Am J Kidney Dis 2015;66:1107–8. 10.1053/j.ajkd.2015.08.017 26363849

[21] Ng DK, Muñoz A, Study C. Assessing bias in GFR estimating equations : improper GFR stratification can yield misleading results. Pediatr Nephrol 2024;39:2139–45. 10.1007/s00467-024-06318-4 38396091 PMC11232499

[22] Delanaye P, Pottel H, Glassock RJ. Americentrism in estimation of GFR equations. Kidney Int 2022;101:856–8. 10.1016/j.kint.2022.02.022 35283173

[23] Shi J, Lindo EG, Baird GS et al. Calculating estimated glomerular filtration rate without the race correction factor: observations at a large academic medical system. Clin Chim Acta 2021;520:16–22. 10.1016/j.cca.2021.05.022 34052206 PMC8286343

[24] KDIGO 2024 Clinical Practice Guideline for the evaluation and management of chronic kidney disease. Kidney Int Suppl 2024;105:S1–314. 10.1016/j.kint.2023.10.018 38490803

[25] Ma Y, Wei L, Yong Z et al. Validation of the European Kidney Function Consortium (EKFC) equation in Chinese adult population: an equation standing on the shoulders of predecessors. Nephron 2024;148:63–73. 10.1159/000531030 37315553

[26] Gama RM, Clery A, Griffiths K et al. Estimated glomerular filtration rate equations in people of self-reported black ethnicity in the United Kingdom: inappropriate adjustment for ethnicity may lead to reduced access to care. PLoS One 2021;16:e0255869. 10.1371/journal.pone.0255869 34383841 PMC8360513

[27] Fu EL, Levey AS, Coresh J et al. Accuracy of GFR estimating equations based on creatinine, cystatin C or both in routine care. Nephrol Dial Transplant 2024;39:694–706. 10.1093/ndt/gfad219 37813817

[28] Nyman U, Grubb A, Larsson A et al. The revised Lund-Malmö GFR estimating equation outperforms MDRD and CKD-EPI across GFR, age and BMI intervals in a large Swedish population. Clin Chem Lab Med 2014;52:815–24. 10.1515/cclm-2013-0741 24334413

[29] Cachat F, Combescure C, Cauderay M et al. A systematic review of glomerular hyperfiltration assessment and definition in the medical literature. Clin J Am Soc Nephrol 2015;10:382–9. 10.2215/CJN.03080314 25568216 PMC4348676

[30] Pottel H, Adebayo OC, Nkoy AB et al. Glomerular hyperfiltration: part 1–defining the threshold—is the sky the limit? Pediatr Nephrol 2023;38:2523–7. 10.1007/s00467-022-05827-4 36459244

[31] Coresh J, Eknoyan G, Levey AS. Estimating the prevalence of low glomerular filtration rate requires attention to the creatinine assay calibration. J Am Soc Nephrol 2002;13:2811–6. 10.1097/01.ASN.0000037420.89149.C9 12397055

[32] Piéroni L, Delanaye P, Boutten A et al. A multicentric evaluation of IDMS-traceable creatinine enzymatic assays. Clin Chim Acta 2011;412:2070–5. 10.1016/j.cca.2011.07.012 21803031

[33] Stevens LA, Manzi J, Levey AS et al. Impact of creatinine calibration on performance of GFR estimating equations in a pooled individual patient database. Am J Kidney Dis 2007;50:21–35. 10.1053/j.ajkd.2007.04.004 17591522

[34] Levey AS, Stevens LA, Schmid CH et al. A new equation to estimate glomerular filtration rate. Ann Intern Med 2009;150:604–12. 10.7326/0003-4819-150-9-200905050-00006 19414839 PMC2763564

[35] Levey AS, Coresh J, Greene T et al. Expressing the modification of diet in renal disease study equation for estimating glomerular filtration rate with standardized serum creatinine values. Clin Chem 2007;53:766–72. 10.1373/clinchem.2006.077180 17332152

