# Supplementary material for: Estimating glomerular filtration in young people
Source: Clin Kidney J. 2024 Aug 28;17(9):sfae261. doi: 10.1093/ckj/sfae261 (PMC11418036; doi:10.1093/ckj/sfae261)
Supplement: sfae261_Supplemental_File [file sfae261_supplemental_file.docx]

**Estimating glomerular filtration in young people**

**Table S1: Creatinine-based equations**

| Name | Age | Sex |  | eGFR-Equation |
| --- | --- | --- | --- | --- |
| CKD-EPI (1) | ≥ 18 | Female | SCr ≤ 0.70 | 143 x (SCr/0.70)^-0.241^ x 0.9938^Age^ |
|  |  |  | SCr > 0.70 | 143 x (SCr/0.70)^-1.200^ x 0.9938^Age^ |
|  |  | Male | SCr ≤ 0.90 | 142 x (SCr/0.90)^-0.302^ x 0.9938^Age^ |
|  |  |  | SCr > 0.90 | 142 x (SCr/0.90)^-1.200^ x 0.9938^Age^ |
| EKFC (2) | 2 - 40 |  | SCr/Q < 1.0 | 107.3 x (SCr/Q)^-0.322^ |
|  |  |  | SCr/Q ≥ 1.0 | 107.3 x (SCr/Q)^-1.132^ |
|  | > 40 |  | SCr/Q < 1.0 | 107.3 x (SCr/Q)^-0.322^ x 0.990^(Age-40)^ |
|  |  |  | SCr/Q ≥ 1.0 | 107.3 x (SCr/Q)^-1.132^ x 0.990^(Age-40)^ |
| r-LMR (3) | 2-17 |  |  | *e*^X – 0.0158 × 18 + 0.438 × ln(18)^ |
|  |  |  | SCr_18_/Q <2.33 | *X* = 4.3087 – 0.7623 × SCr_18_/Q |
|  |  |  | SCr_18_/Q ≥2.33 | *X* = 3.3145 – 0.9260 × ln(SCr_18_/Q) |
|  | ≥ 18 |  |  | *e*^X – 0.0158 × Age + 0.438 × ln(Age)^ |
|  |  |  | SCr/Q <2.33 | *X* = 4.3087 – 0.7623 × SCr/Q |
|  |  |  | SCr/Q ≥2.33 | *X* = 3.3145 – 0.9260 × ln(SCr/Q) |

CKD-EPI: Chronic Kidney Disease Epidemiology, EKFC: European Kidney Function Consortium, r-LMR: re-expressed Lund-Malmö, SCr: Serum creatinine.

Q = rescaling factor for creatinine

**EKFC:** To make a more continuous transition from children to adults using the EKFC equation, the Q-values for children, adolescents and young adults (up to 25 years) can be calculated from (note that the Q-value obtained from these equations is expressed in µmol/L, divide by 88.4 to obtain creatinine in mg/dL):

- Men, age ≤ 25: ln(Q) = 3.200 + 0.259 x age - 0.543 x log(age) - 0.00763 x age² + 0.0000790 x age³
- Women, age ≤ 25 ln(Q) = 3.080 + 0.177 x age - 0.223 x log(age) - 0.00596 x age² + 0.0000686 x age³

For European subjects, age > 25 years, use

- Men: Q = 0.90 mg/dL
- Women: Q = 0.70 mg/dL (see (4,5) for Q values in other populations)

For US subjects, age > 25 years, use race-free Q values

- Men: Q = 0.97 mg/dL
- Women: Q = 0.73 mg/dL (see (4,5) for Q values in other populations)

**r-LMR**: r-LMR is a simplification of the original re-expressed equation in so far as the coefficients for females and males are fairly similar why a non-sex specific equation was created by averaging their coefficients (3).To make a continuous transition from children to adults using the r-LMR equation, established creatinine growth curve below (REF) are used to recalculate childhood levels of creatinine to corresponding levels at the age of 18 years:

- Boys: ln (sCr_18_) = ln(sCr) + 0.259 × (18 - *Age*) - 0.543 × ln(18 / *Age*) - 0.00763 × (18^2^ - *Age*^2^) + 0.0000790 × (18^3^ - *Age*^3^)
- Girls: ln (sCr_18_) = ln(sCr) + 0.177 × (18 - *Age*) - 0.223 × ln(18 / *Age*) - 0.00596 × (18^2^ - *Age*^2^) + 0.0000686 × (18^3^ - *Age*^3^)

The adult Q-values according to above (e.g. 0.7/0.9 mg/dL for white Europeans) are then used irrespectively of age in the r-LMR equation.

**Table S2: Characteristics of the cohorts**

**EUROPE**

| **Center** | **Country** | **Cohort** | **N** | **ClearanceMethod**  **P=Plasma**  **R=Renal** | **Exogenous marker** | **Age**  **(mean ± SD)**  **(year)** | **mGFR**  **(mean ± SD)**  **(mL/min/1.73m²)** | **Serum**  **creatinine**  **(mg/dL)** | **% Females** |
| --- | --- | --- | --- | --- | --- | --- | --- | --- | --- |
| Lyon | France | Referrals* | 1493 | R/P | inulin / iohexol | 20.8±2.1 | 88.6±29.7 | 0.96±0.43 | 48.6 |
| Örebro | Sweden | Referrals* | 107 | P | iohexol | 21.4±2.2 | 91.8±26.2 | 1.09±0.57 | 58.8 |
| Saint-Etienne | France | HIV-study^3^ | 3 | R | inulin | 23.7±1.5 | 101.8±7.1 | 0.61±0.14 | 66.7 |
| Stockholm | Sweden | Referrals* | 47 | P | iohexol | 18.2±0.2 | 93.0±21.9 | 0.92±0.28 | 38.3 |
| Amsterdam | The Netherlands | CAPA Study (6)+ Referrals* | 48 | P | Inulin | 18.7±0.9 | 90.5±23.3 | 0.97±0.37 | 25.0 |
| France MC | France | Kidney Donor Study(7) | 51 | P/R | Iohexol / inulin / ^51^Cr-EDTA | 22.7±1.7 | 105.5±15.5 | 0.87±0.18 | 37.3 |
| Leuven | Belgium | Referrals* | 21 | P | ^51^Cr-EDTA | 19.1±1.2 | 86.5±20.5 | 0.72±0.38 | 47.6 |
| Lund | Sweden | CAPA Study (6) | 120 | P | iohexol | 20.2±2.5 | 90.2±29.2 | 0.98±0.62 | 36.7 |
| **All** |  |  | **1892** |  |  | **20.7±2.1** | **89.5±28.9** | **0.96±0.44** | **46.3** |

*Referrals = referred for plasma or renal clearance measurement on clinical grounds

mGFR: measured GFR

**US**

| **Center** | **Cohort** | **N** | **ClearanceMethod**  **P=Plasma**  **R=Renal** | **Exogenous marker** | **Age**  **(mean ± SD)**  **(year)** | **mGFR**  **(mean ± SD)**  **(mL/min/1.73m²)** | **Serum**  **creatinine**  **(mg/dL)** | **% Females** | **% Black**  **(versus non-Black)** |
| --- | --- | --- | --- | --- | --- | --- | --- | --- | --- |
| AASK | (8) | 16 | R | iothalamate | 22.9±1.9 | 54.4±31.4 | 1.99±1.01 | 31.3 | 100 |
| ALTOLD | (9) | 16 | P | iohexol | 22.4±1.4 | 110.4±15.5 | 0.85±0.15 | 43.8 | 6.3 |
| CRIC | (10) | 8 | R | iothalamate | 23.3±1.4 | 40.0±19.9 | 2.32±0.98 | 37.5 | 50.0 |
| CRISP | (11) | 45 | R | iothalamate | 21.5±2.6 | 107.6±22.8 | 0.95±0.24 | 48.9 | 13.3 |
| DCCT/EDIC | (12) | 141 | R | iothalamate | 23.4±1.4 | 117.6±17.0 | 0.77±0.13 | 58.9 | 0 |
| Mayo Clinic | Referrals*(13) | 186 | R | iothalamate | 21.9±2.2 | 72.9±36.8 | 1.36±0.96 | 45.7 | 1.6 |
| MDRD | (14) | 61 | R | iothalamate | 22.7±2.1 | 39.2±23.3 | 2.33±1.15 | 42.6 | 11.5 |
| PERL | (15) | 1 | P | iohexol | 23 | 93.8 | 0.97 | 0 | 0 |
| **All** |  | **474** |  |  | 22.5±2.1 | **85.3±39.5** | **1.29±0.93** | **48.7** | **7.8** |

*Referrals = referred for plasma or renal clearance measurement on clinical grounds

AASK: African American Study of Kidney Disease and Hypertension, ALTOLD: Assessing Long Term Outcomes in Living Kidney Donors, CRIC: Chronic Renal Insufficiency Cohort, CRISP: Consortium for Radiologic Imaging Studies of Polycystic Kidney Disease, DCCT/EDIC: Diabetes Control and Complications Trial / Epidemiology of Diabetes Interventions and Complications, MDRD: Modified Diet in Renal Disease study (MDRD), mGFR: measured GFR, PERL: Preventing Early Renal Loss in Diabetes.

**Table S3: Performance of the CKD-EPI and EKFC equations in 18 to 25 years old males and females**

|  | **n** | **Bias** | | | **IQR** | | | **P30** | | |
| --- | --- | --- | --- | --- | --- | --- | --- | --- | --- | --- |
| **Europe** |  | CKD-EPI | EKFC | r-LMR | CKD-EPI | EKFC | r-LMR | CKD-EPI | EKFC | r-LMR |
| Males | 1016 | 18.47 [16.91;19.36] | **1.97**  **[1.06;3.32]** | **-1.78**  **[-2.60;0.67]** | 22.2  [7.3;29.4] | 20.9  [-9.0;11.9] | 21.1  [-13.6;7.5] | 65.0  [62.0;67.9] | **84.4**  **[82.1;86.6]** | **86.6**  **[84.5;88.7]** |
| Females | 876 | 16.46  [15.10;17.61] | **2.47**  **[1.51;3.06]** | **-4.84**  **[-6.14;2.18]** | 25.0  [4.5;29.5] | 23.0  [-8.4;14.6] | 23.0  [-15.3;7.7] | 66.0  [62.8;69.1] | **84.5**  **[82.1;86.9]** | **87.9**  **[85.7;90.1]** |
| **USA** |  | | | | | | | | | |
| Males | 243 | 8.28  [4.81;11.00] | **3.04**  **[0.80;4.63]** | **-0.20**  **[-2.35;2.41]** | 26.5  [-3.3;23.2] | 24.9  [-11.1;13.8] | 25.4  [-16.1;9.2] | 70.0  [64.2;75.7] | 77.0  [71.7;82.2] | 78.6  [73.4;83.8**]** |
| Females | 231 | 6.04  [3.61;8.46] | -2.30  [-4.11;-0.05] | -6.64  [-10.27;-3.57] | 18.9  [-2.8;16.0] | 19.6  [-12.4;7.2] | 24.1  [-20.5;3.6] | 78.4  [73.0;83.7] | 81.8  [76.8;86.8] | 82.3  [77.3;87.2**]** |

CKD-EPI: Chronic Kidney Disease Epidemiology collaboration, EKFC: European Kidney Function Consortium, IQR: interquartile range (of the bias), P30: accuracy within 30%, r-LMR: re-expressed Lund-Malmö, NA: not available.

Bias and IQR are expressed in mL/min/1.73m². P30 is expressed in %. Significant better results are in bold.

**Table S4: Performance of the CKD-EPI and EKFC equations in 18 to 25 years old according to estimated or measured GFR**

|  | **n** | | | **Bias** | | | **IQR** | | | **P30** | | |
| --- | --- | --- | --- | --- | --- | --- | --- | --- | --- | --- | --- | --- |
| All results in mL/min/1.73m² | CKD-EPI | EKFC | r-LMR | CKD-EPI | EKFC | r-LMR | CKD-EPI | EKFC | r-LMR | CKD-EPI | EKFC | r-LMR |
| **Europe** |  | | | | | | | | | | | |
| eGFR<30 | 21 | 26 | 31 | 4.19  [0.78;4.49] | 1.86  [-1.64;2.87] | 1.17  [-1.29;1.30] | 4.4  [0.8;5.2] | 6.0  [-2.7;3.2] | 4.1  [-2.5;1.7] | 90.5  [77.9;103.0] | 73.1  [56.0;90.1] | 87.1  [75.3;98.9] |
| eGFR [30-45[ | 44 | 61 | 82 | 8.55  [5.13;9.64] | **3.35**  **[1.10;4.61]** | **-1.17**  **[-2.57;0.54]** | 7.1  [3.5;10.6] | 8.5  [-0.5;8.1] | 8.2  [-5.2;3.1] | 56.8  [42.2;71.5] | 78.7  [68.4;89.0] | **84.1**  **[76.2;92.1]** |
| eGFR [45-60[ | 79 | 153 | 153 | 7.91  [7.32;9.82] | **1.83**  **[-0.60;3.44]** | **-1.90**  **[-3.85;0.68]** | 9.2  [4.4;13.6] | 17.2  [-5.8;11.4] | 19.3  [-9.3;10.0] | 67.1  [56.7;77.5] | 71.9  [64.8;79.0] | 77.1  [70.5;83.8**]** |
| eGFR [60-90[ | 379 | 527 | 715 | 13.75  [11.32;15.34] | **2.45**  **[-0.09;3.72]** | **-2.76**  **[-4.86;-1.30]** | 22.2  [1.4;23.6] | 24.2  [-13.3;10.9] | 24.2  [-17.0;7.2] | 58.3  [53.3;63.3] | **80.1**  **[76.7;83.5]** | **85.5**  **[82.9;88.0]** |
| eGFR [90-120[ | 500 | 1009 | 804 | 16.88  [15.46;17.61] | **1.91**  **[1.03;2.69]*** | **-5.73**  **[-6.76;-3.42]** | 22.9  [4.7;27.5] | 21.2  [-7.9;13.3] | 21.9  [-14.7;7.1] | 70.0  [66.0;74.0] | **89.8**  **[87.9;91.7]** | **92.0**  **[90.2;93.9]** |
| eGFR ≥ 120 | 869 | 116 | 107 | 22.84  [21.10;24.41] | **6.26**  **[2.19;12.05]** | **7.96**  **[5.53;13.93]** | 24.3  [11.4;35.7] | 37.8  [-15.0;22.8] | 39.5  [-13.6;25.8] | 65.6  [62.4;68.8] | **79.3**  **[71.9;86.7]** | **79.4**  **[71.8;87.1]** |
| **Europe** |  | | | | | | | | | | | |
| mGFR<30 | 43 | | | 8.95  [6.23;13.23] | 6.28  [3.01;8.72] | **2.97**  **[1.60;5.32]** | 12.1  [4.5;16.6] | 10.9  [2.2;13.1] | 6.2  [1.2;7.4] | 39.5  [24.9;54.1] | 53.5  [38.6;68.4] | **76.2**  **[63.3;89.1]** |
| mGFR [30-45[ | 107 | | | 20.55  [13.13;23.56] | 12.55  [7.24;16.25] | **10.85**  **[2.58;12.62]** | 19.3  [8.8;28.1] | 16.2  [3.3;19.6] | 18.2  [-1.4;16.7] | 32.7  [23.8;41.6] | 43.0  [33.6;52.4] | 49.1  [39.6;58.5] |
| mGFR [45-60[ | 169 | | | 19.54  [15.76;22.39] | **9.61**  **[8.17;12.04]** | **8.09**  **[5.38;9.97]** | 22.8  [8.2;31.0] | 16.7  [1.8;18.6] | 17.9  [-1.8;16.2] | 40.2  [32.8;47.6] | **68.6**  **[61.6;75.6]** | **74.0**  **[67.3;80.6]** |
| mGFR [60-90[ | 550 | | | 24.62  [22.92;26.98] | **8.97**  **[7.76;10.35]** | **4.84**  **[3.14;5.57]*** | 27.2  [13.0;40.2] | 21.6  [-0.5;21.1] | 16.4  [-2.9;13.5] | 45.5  [41.3;49.6] | **78.4**  **[74.9;81.8]** | **86.9**  **[84.1;89.7]*** |
| mGFR [90-120[ | 767 | | | 18.25  [17.15;19.54] | **-0.46**  **[-1.35;0.68]*** | **-8.85**  **[-9.57;-7.96]** | 19.2  [9.3;28.5] | 17.0  [-8.6;8.4] | 17.9  [-16.0;1.9] | 80.2  [77.4;83.0] | **96.7**  **[95.5;98.0]** | **95.4**  **[94.0;96.9]** |
| mGFR ≥ 120 | 256 | | | **0.95**  **[0.14;2.85]** | -16.73  [-18.04;-15.80]* | -23.98  [-25.43;-20.71] | 17.6  [-8.0;9.6] | 14.1  [-25.1;-1.1] | 16.1  [-31.5;-15.3] | **98.8**  **[97.5;100.1]** | 93.4  [90.3;96.4] | 89.8  [86.1;93.5] |
| **USA** |  | | | | | | | | | | | |
| eGFR<30 | 42 | 42 | 55 | 1.38  [0.03;3.62] | 1.63  [-0.12;3.04] | -0.18  [-1.50;2.58] | 6.1  [-0.8;5.3] | 5.7  [-1.1;4.7] | 6.3  [-1.9;4.4] | 71.4  [57.8;85.1] | 71.4  [57.8;85.1] | 74.5  [63.0;86.1] |
| eGFR [30-45[ | 37 | 44 | 38 | 6.44  [4.87;10.5] | 7.26  [2.65;9.50] | 3.18  [-1.98;5.24] | 8.2  [2.9;11.1] | 9.5  [1.2;10.7] | 11.4  [-4.6;6.8] | 59.5  [43.6;75.3] | 54.5  [39.8;69.3] | 81.6  [69.3;93.9] |
| eGFR [45-60[ | 38 | 40 | 35 | 8.86  [2.04;14.35] | 3.69  [-0.20;7.15] | 1.61  [-1.94;6.99] | 18.0  [-0.6;17.3] | 17.2  [-4.4;12.7] | 16.3  [-5.4;11.0] | 55.3  [39.5;71.1] | 70.0  [55.8;84.2] | 77.1  [63.2;91.1] |
| eGFR [60-90[ | 58 | 82 | 111 | 6.09  [-1.22;10.31] | -2.26  [-8.97;4.48] | -8.11  [-13.89; -3.26] | 26.5  [-9.0;17.5] | 32.3  [-20.2;12.1] | 30.2  [-24.7;5.5] | 69.0  [57.1;80.9] | 70.7  [60.9;80.6] | 72.1  [63.7;80.4] |
| eGFR [90-120[ | 144 | 245 | 210 | 0.33  [-3.02;4.47] | -3.85  [-7.17;-2.14] | -12.13  [-15.06;-6.50] | 29.0  [-12.7;16.2] | 26.2  [-16.4;9.9] | 28.4  [-23.7;4.7] | 85.4  [79.7;91.2] | 89.0  [85.1;92.9] | 86.7  [82.1;91.3] |
| eGFR ≥ 120 | 155 | 21 | 25 | 16.18  [12.38;20.99] | 13.11  [-3.41;23.60] | 12.31  [-1.03;23.95] | 28.0  [2.7;30.7] | 28.4  [-3.5;25.0] | 26.9  [-2.1;24.8] | 74.2  [67.3;81.1] | 85.7  [70.7;100.7] | 80.0  [64.3;95.7] |
| **USA** |  | | | | | | | | | | | |
| mGFR<30 | 68 | | | 5.97  [2.30;8.59] | 4.98  [2.59;8.54] | 3.63  [1.35;4.82] | 10.8  [0.6;11.4] | 10.3  [0.3;10.6] | 7.8  [-1.0;6.8] | 50.0  [38.1;61.9] | 50.0  [38.1;61.9] | 69.1  [58.1;80.1] |
| mGFR [30-45[ | 33 | | | 8.02  [5.40;14.20] | 7.20  [2.65;10.64] | 2.99  [-1.79;7.83] | 12.0  [3.0 ;15.1] | 10.3  [2.2;12.5] | 11.7  [-2.2;9.5] | 57.6  [40.7;74.4] | 66.7  [50.6;82.8] | 72.7  [57.5;87.9] |
| mGFR [45-60[ | 37 | | | 8.46  [4.52;19.43] | 5.25  [1.80;14.82] | 4.03  [-0.19;12.23] | 25.7  [2.0;27.7] | 20.8  [-0.2;20.6] | 20.8  [-1.9;18.8] | 59.5  [43.6;75.3] | 62.2  [46.5;77.8] | 62.2  [46.5;77.8] |
| mGFR [60-90[ | 80 | | | 18.56  [11.19;23.82] | 9.21  [4.89;13.99] | 5.12  [2.34;8.40] | 28.2  [1.7;29.9] | 21.5  [-2.9;18.6] | 17.9  [-4.7;13.2] | 61.3  [50.6;71.9] | **81.3**  **[72.7;89.8]** | **86.3**  **[78.7;93.8]** |
| mGFR [90-120[ | 163 | | | 11.99  [9.41;14.55] | **-2.18**  **[-3.84;2.09]*** | -8.05  [-11.52;-4.38] | 23.3  [0.1;23.3] | 18.5  [-8.8;9.7] | 21.8  [-17.7;4.1] | 87.1  [82.0;92.3] | **97.5**  **[95.2;99.9]** | **95.7**  **[92.6;98.8**] |
| mGFR ≥ 120 | 93 | | | **-11.24**  **[-18.20;-9.27]** | -25.12  [-30.92;-20.86] | -31.49  [-35.97;-28.53] | 24.3  [-25.8;-1.5] | 20.7  [-37.5;-16.8] | 22.0  [-43.7;-21.7] | **91.4**  **[85.7;97.1]** | 78.5  [70.1;86.8] | 66.7  [57.1;76.2] |

CKD-EPI: Chronic Kidney Disease Epidemiology, EKFC: European Kidney Function Consortium, eGFR: estimated glomerular filtration rate, IQR: interquartile range (of the bias), mGFR: measured glomerular filtration rate, P30: accuracy within 30%, r-LMR: re-expressed Lund-Malmö. Bias and IQR are expressed in mL/min/1.73m². P30 is expressed in %. Significant better results are in bold. * if r-LMR is better than EKFC or EKFC better than r-LMR.

**Table S5: Performance of the CKD-EPI and EKFC equations in 18 to 25 years old according to body mass index in European cohorts**

|  | **n** | **Bias** | | | **IQR** | | | **P30** | | |
| --- | --- | --- | --- | --- | --- | --- | --- | --- | --- | --- |
| All results in mL/min/1.73m² |  | CKD-EPI | EKFC | r-LMR | CKD-EPI | EKFC | r-LMR | CKD-EPI | EKFC | r-LMR |
| **Europe** |  | | | | | | | | | |
| BMI<18 kg/m² | 268 | 28.10  [25.85;31.42] | **13.04**  **[10.82;16.25]** | **6.13**  **[3.72; 7.97]*** | 22.7  [18.0;40.7] | 21.9  [2.7;24.6] | 23.3  [-5.5; 17.8] | 43.3  [37.4;49.2] | **70.1**  **[64.7;75.6]** | **73.0**  **[67.7; 78.4]** |
| BMI [18-25[ kg/m² | 1295 | 16.54  [15.61;17.44] | **3.05**  **[2.06;3.71]** | **-4.07**  **[-4.97; -2.60]** | 23.2  [5.4;28.6] | 20.7  [-7.9;12.8] | 21.5  [-15.0; 6.6] | 67.7  [65.2;70.3] | **85.6**  **[83.7;87.5]** | **89.3**  **[87.6; 91.0]*** |
| BMI [25-30[ kg/m² | 247 | 13.45  [11.80;16.06] | **0.56**  **[-1.03;2.28]*** | **-5.89**  **[-8.64; -2.57]** | 19.7  [4.5;24.1] | 17.8  [-6.8;11.0] | 18.9  [-14.7; 4.2] | 74.9  [69.5;80.3] | **89.9**  **[86.1;93.6]** | **92.2**  **[88.8; 95.6]** |
| BMI ≥ 30 kg/m² | 82 | 17.68  [12.22;20.61] | **3.16**  **[-1.74;8.48]** | **-5.81**  **[-12.50; 3.18]** | 22.6  [5.0;27.6] | 26.4  [-11.0;15.4] | 30.9  [-20.2; 10.7] | 63.4  [53.0;73.8] | **74.4**  **[64.9;83.8]** | **85.4**  **[77.7; 93.0]** |

BMI: Body mass index, CKD-EPI: Chronic Kidney Disease Epidemiology, EKFC: European Kidney Function Consortium, IQR: interquartile range (of the bias), P30: accuracy within 30%, r-LMR: re-expressed Lund-Malmö. Bias and IQR are expressed in mL/min/1.73m². P30 is expressed in %. Significant better results are in bold. * if r-LMR is better than EKFC or EKFC better than r-LMR.

**Table S6: Performance of the EKFC equations in 18 to 25 years old using the polynomial versus the fixed Q-values**

|  | **n** | | **Bias** | | **IQR** | | **P30** | |
| --- | --- | --- | --- | --- | --- | --- | --- | --- |
| All results in mL/min/1.73m² | EKFC | EKFC Fixed | EKFC | EKFC Fixed | EKFC | EKFC Fixed | EKFC | EKFC Fixed |
| **Europe** |  |  |  |  |  |  |  |  |
| All | 1892 | | **2.28**  **[1.59;2.91]** | 3.57  [3.05;4.39] | 21.4  [-8.7;12.7] | 21.5  [-7.3;14.2] | 84.4  [82.8;86.0] | 83.5  [81.8;85.2] |
| mGFR<30 | 43 | | 6.28  [3.01;8.72] | 6.54  [4.16;10.07] | 10.9  [2.2;13.1] | 10.1  [3.4;13.4] | 53.5  [38.6;68.4] | 55.8  [41.0;70.7] |
| mGFR [30-45[ | 107 | | 12.55  [7.24;16.25] | 15.43  [7.90;16.53] | 16.2  [3.3;19.6] | 16.7  [3.6;20.3] | 43.0  [33.6;52.4] | 41.1  [31.8;50.4] |
| mGFR [45-60[ | 169 | | 9.61  [8.17;12.04] | 11.37  [8.60;13.90] | 16.7  [1.8;18.6] | 17.9  [2.7;20.6] | 68.6  [61.6;75.6] | 65.7  [58.5;72.8] |
| mGFR [60-90[ | 550 | | 8.97  [7.76;10.35] | 11.56  [10.06;12.63] | 21.6  [-0.5;21.1] | 20.8  [2.6;23.4] | 78.4  [74.9;81.8] | 75.3  [71.7;78.9] |
| mGFR [90-120[ | 767 | | -0.46  [-1.35;0.68] | 1.16  [-0.10;2.05] | 17.0  [-8.6;8.4] | 17.0  [-6.7;10.3] | 96.7  [95.5;98.0] | 96.7  [95.5;98.0] |
| mGFR ≥ 120 | 256 | | -16.73  [-18.04;-15.80] | -15.94  [-17.57;-14.56] | 14.1  [-25.1;-1.1] | 14.4  [-24.2;-9.7] | 93.4  [90.3;96.4] | 95.7  [93.2;98.2] |
| **USA** |  |  |  |  |  |  |  |  |
| All | 474 | | 0.37  [-1.69;2.24] | 1.45  [-0.54;3.38] | 22.3  [-11.8;10.5] | 22.6  [-10.8;11.9] | 79.3  [75.7;83.0] | 78.7  [75.0;82.4] |
| mGFR<30 | 68 | | 4.98  [2.59;8.54] | 5.92  [3.19;9.15] | 10.3  [0.3;10.6] | 10.8  [0.8;11.6] | 50.0  [38.1;61.9] | 48.5  [36.7;60.4] |
| mGFR [30-45[ | 33 | | 7.20  [2.65;10.64] | 7.05  [3.83;12.53] | 10.3  [2.2;12.5] | 11.1  [2.8;14.0] | 66.7  [50.6;82.8] | 57.6  [40.7;74.4] |
| mGFR [45-60[ | 37 | | 5.25  [1.80;14.82] | 6.98  [3.70;15.70] | 20.8  [-0.2;20.6] | 23.6  [1.8;25.4] | 62.2  [46.5;77.8] | 59.5  [43.6;75.3] |
| mGFR [60-90[ | 80 | | 9.21  [4.89;13.99] | 12.09  [8.43;16.92] | 21.5  [-2.9;18.6] | 22.9  [-1.2;21.7] | 81.3  [72.7;89.8] | 78.8  [69.8;87.7] |
| mGFR [90-120[ | 163 | | -2.18  [-3.84;2.09] | 0.17  [-2.77;3.53] | 18.5  [-8.8;9.7] | 18.1  [-8.2;9.9] | 97.5  [95.2;99.9] | 96.9  [94.3;99.6] |
| mGFR ≥ 120 | 93 | | -25.12  [-30.92;-20.86] | -23.67  [-29.07;-20.62] | 20.7  [-37.5;-16.8] | 21.8  [-37.0;-15.2] | 78.5  [70.1;86.8] | 83.9  [76.4;91.3] |

EKFC: European Kidney Function Consortium, IQR: interquartile range (of the bias), mGFR: measured glomerular filtration rate, P30: accuracy within 30%. Bias and IQR are expressed in mL/min/1.73m². P30 is expressed in %. Significant better results are in bold.

Polynomial Q-values can be calculated from (note that the Q-value obtained from these equations is expressed in µmol/L):

- Men, age ≤ 25: ln(Q) = 3.200 + 0.259 x age - 0.543 x log(age) - 0.00763 x age² + 0.0000790 x age³
- Women, age ≤ 25 ln(Q) = 3.080 + 0.177 x age - 0.223 x log(age) - 0.00596 x age² + 0.0000686 x age³

Q can be obtained in mg/dL using exp(Q)/88.4

Fixed Q values:

For White European subjects, age > 25 years, use

- Men: Q = 0.90 mg/dL
- Women: Q = 0.70 mg/dL (see (4,5) for Q values in other populations)

For US subjects, age > 25 years, use race-free Q-value

- Men: Q = 0.97 mg/dL
- Women: Q = 0.73 mg/dL (see (4,5) for Q values in other populations)

**Figure S1-4: Theoretical comparison of CKD-EPI 2021 and EKFC at 18 and 35 years**

The EKFC and CKD-EPI equation can be compared at the same age, for the same sex, and for all possible values of SCr varying from low to high. This is shown in the figures below.

| 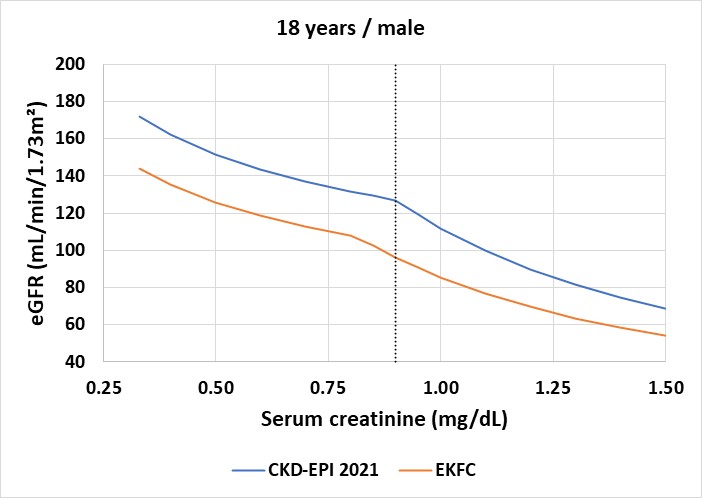 | 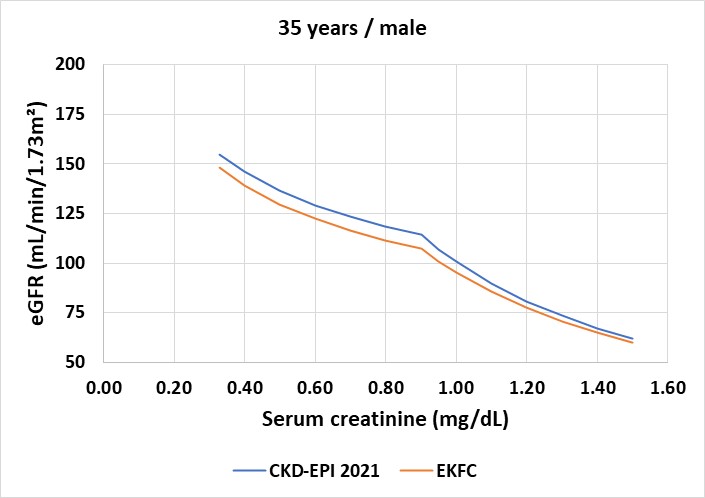 |
| --- | --- |
| 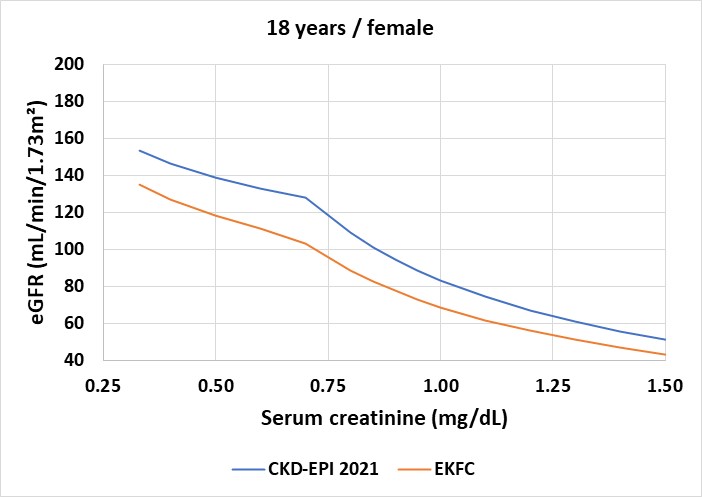 | 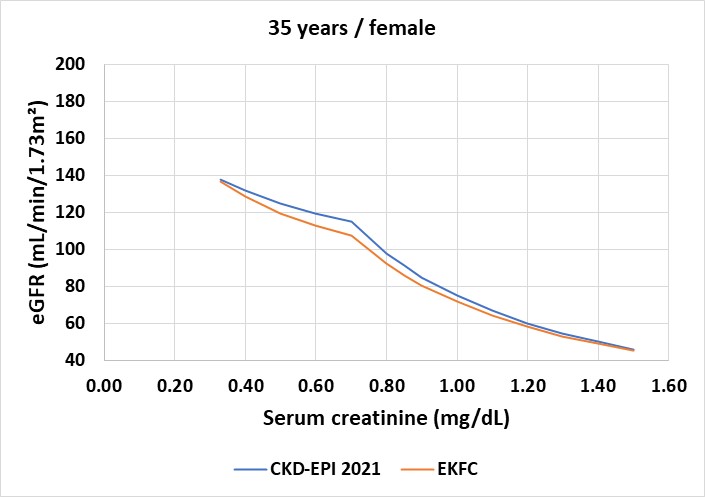 |

At 18 years, there is a systematic difference between CKD-EPI and EKFC, with CKD-EPI systematically predicting higher eGFR-values than EKFC, for all values of SCr.

At 35 years, both equations are much closer to each other, with nearly no difference between them.

The difference at 18 years is explained by the design of the equation, which is totally different at young adult ages. EKFC takes into account the different behavior of GFR vs age at young adult age, compared to older adult age, by including two splines switching at the age of 40 years. The first spline assumes that GFR remains more or less constant over the 18-40 year age-range and the second spline, starting at 40 years, assumes a gradual decline with age. CKD-EPI, on the contrary, assumes the same decline rate in the + 18 year age-range. This is due to the fact that CKD-EPI used multilinear regression of log(mGFR) versus log(SCr), age and sex over the 18-75 year age-range as the statistical methodology to develop their equation. In other words, the basic mathematical relationship between mGFR and age is fundamentally different between EKFC and CKD-EPI. As EKFC has been developed as a full age range equation (that is, also applicable for children), the EKFC-equation takes into account the transition from childhood to adulthood and this is totally absent in the development of the CKD-EPI equation. Therefore, the mathematical underlying assumptions were fundamentally wrong during the development of the CKD-EPI equation.

**References**

1. Inker LA, Eneanya ND, Coresh J et al. New Creatinine- and Cystatin C–Based Equations to Estimate GFR without Race. N Engl J Med 2021; 385: 1737–1749.

2. Pottel H, Björk J, Courbebaisse M et al. Development and Validation of a Modified Full Age Spectrum Creatinine-Based Equation to Estimate Glomerular Filtration Rate. A Cross-sectional Analysis of Pooled Data. Ann Intern Med 2021; 174: 183–191.

3. Nyman U, Björk J, Delanaye P et al. Rescaling creatinine makes GFR estimation equations generally applicable across populations - validation results for the Lund-Malmö equation in a French cohort of sub-Saharan ancestry. Clin Chem Lab Med 2023; 62: 421–427.

4. Delanaye P, Vidal-Petiot E, Björk J et al. Performance of creatinine-based equations to estimate glomerular filtration rate in White and Black populations in Europe, Brazil, and Africa. Nephrol Dial Transplant 2023; 38: 106–118.

5. Ma Y, Wei L, Yong Z et al. Validation of the European Kidney Function Consortium (EKFC) equation in Chinese adult population: an equation standing on the shoulders of predecessors. Nephron 2023; In process.

6. Björk J, Grubb A, Larsson A et al. Accuracy of GFR estimating equations combining standardized cystatin C and creatinine assays: a cross-sectional study in Sweden. Clin Chem Lab Med 2015; 53: 403–414.

7. Gaillard F, Courbebaisse M, Kamar N et al. The age-calibrated measured glomerular filtration rate improves living kidney donation selection process. Kidney Int 2018; 94: 616–624.

8. Lewis J, Agodoa L, Cheek D et al. Comparison of cross-sectional renal function measurements in African Americans with hypertensive nephrosclerosis and of primary formulas to estimate glomerular filtration rate. Am J Kidney Dis 2001; 38: 744–753.

9. Kasiske BL, Anderson-Haag TL, Duprez DA et al. A prospective controlled study of metabolic and physiologic effects of kidney donation suggests that donors retain stable kidney function over the first nine years. Kidney Int 2020; 98: 168–175.

10. Hsu C, Yang W, Parikh R V. et al. Race, Genetic Ancestry, and Estimating Kidney Function in CKD. N Engl J Med 2021; 385: 1750–1760.

11. Rule AD, Torres VE, Chapman AB et al. Comparison of methods for determining renal function decline in early autosomal dominant polycystic kidney disease: the consortium of radiologic imaging studies of polycystic kidney disease cohort. J Am Soc.Nephrol 2006; 17: 854–862.

12. de Boer IH, Sun W, Cleary PA et al. Longitudinal Changes in Estimated and Measured GFR in Type 1 Diabetes. J Am Soc Nephrol 2014; 25: 810–818.

13. Zhang X, Rule AD, McCulloch CE, Lieske JC, Ku E, Hsu CY. Tubular secretion of creatinine and kidney function: An observational study. BMC Nephrol 2020; 21: 1–9.

14. Levey AS, Bosch JP, Lewis JB, Greene T, Rogers N, Roth D. A more accurate method to estimate glomerular filtration rate from serum creatinine: a new prediction equation. Modification of Diet in Renal Disease Study Group. Ann Intern Med 1999; 130: 461–470.

15. Doria A, Galecki AT, Spino C et al. Serum Urate Lowering with Allopurinol and Kidney Function in Type 1 Diabetes. N Engl J Med 2020; 382: 2493–2503.
